# The role of clonal progression leading to the development of therapy-related myeloid neoplasms

**DOI:** 10.1007/s00277-024-05803-y

**Published:** 2024-07-20

**Authors:** Luca Guarnera, Maria Rosaria Pascale, Hajro Hajrullaj, Antonio Cristiano, Flavia Mallegni, Angelo Onorato, Maria Teresa Voso, Emiliano Fabiani

**Affiliations:** 1 Department of Biomedicine and Prevention, PhD in Immunology, Molecular Medicine and Applied Biotechnology, University of Rome Tor Vergata, Rome, Italy; 2 Department of Translational Hematology & Oncology Research, Taussig Cancer Institute, Cleveland Clinic, Cleveland, OH 44114 USA; 3 Transfusion Medicine Unit, Cardarelli Hospital, 86100 Campobasso, Italy; 4 Department of Biomedicine and Prevention, University of Rome Tor Vergata, Rome, Italy; 5 Department of Biomedicine and Prevention, PhD in Medical-Surgical Biotechnologies and Translational Medicine, University of Rome Tor Vergata, Rome, Italy; 6 Neuro-Oncohematology Unit, Istituto Di Ricovero E Cura a Carattere Scientifico (IRCCS) Fondazione Santa Lucia, Rome, Italy; 7 UniCamillus-Saint Camillus International University of Health Sciences, Rome, Italy

**Keywords:** Therapy-related Myeloid Neoplasms, Myeloid neoplasms post cytotoxic therapy, Clonal Hematopoiesis of Indeterminate Potential, TP53

## Abstract

Therapy-related myeloid neoplasms (t-MN) are characterized by aggressive features and a dismal prognosis. Recent evidence suggests a higher incidence of t-MN in individuals harboring clonal hematopoiesis of indeterminate potential (CHIP). In order to gain insight into CHIP-driven malignant progression, we gathered data from ten published reports with available detailed patient characteristics at the time of primary malignancy and t-MN development. Detailed clinical and molecular information on primary malignancy and t-MN were available for 109 patients: 43% harbored at least one somatic mutation at the time of the primary malignancy. *TET2* and *TP53* mutations showed an increasing variant allele frequency from CHIP to t-MN. *ASXL1*-associated CHIP significantly correlated with the emergence of *TET2* and *CEBPA* mutations at t-MN, as well as *U2AF1*-driven *CHIP* with *EZH2* mutation and both *IDH2* and *SRSF2*-driven CHIP with *FLT3* mutation. *DNMT3A*-driven CHIP correlated with a lower incidence of *TP53* mutation at t-MN. In contrast, *TP53*-driven CHIP correlated with a complex karyotype and a lower tendency to acquire new mutations at t-MN. Patients with multiple myeloma as their first malignancy presented a significantly higher rate of *TP53* mutations at t-MN. The progression from CHIP to t-MN shows different scenarios depending on the genes involved. A deeper knowledge of CHIP progression mechanisms will allow a more reliable definition of t-MN risk.

**Supplementary Information:**

The online version contains supplementary material available at 10.1007/s00277-024-05803-y.

## Introduction

Therapy-related myeloid neoplasms (t-MN) or myeloid neoplasm post cytotoxic therapy (MN-pCT), as defined according to the 5th edition of WHO Myeloid Neoplasia Classification, encompasses conditions such as acute myeloid leukemia (AML), myelodysplastic syndromes (MDS), and myelodysplastic/myeloproliferative neoplasms (MDS/MPN) with a documented history of chemo/radiotherapy as treatment for an unrelated condition [1, 2]. Although “post cytotoxic therapy” or “therapy-related” (as per International Consensus Classification, ICC, nomenclature [3]) have lost their autonomous entity status and are disease attributes in the new classifications, the recognition of this feature remains of major importance.

t-MN are characterized by poor prognosis and a specific karyotype/genetic signature, enriched in complex cytogenetic abnormalities and high-risk mutations, especially *TP53*, detected in approximately 20–40% of cases [4]. The precise mechanisms leading to malignancy development, involving complex microenvironment interactions, inherited predisposition, drug genotoxicity and clonal selection, are still the object of extensive study [4, 5]. Clonal hematopoiesis of indeterminate potential (CHIP) has been considered one of the main risk factors and has been identified at the time of the primary cancer diagnosis in 30–70% of patients developing a t-MN, potentially being the *primum movens* of clonal evolution toward malignancy [6–9]. Most individuals with CHIP, defined as the presence of mutations in myeloid genes at a variant allele frequency (VAF) ≥ 2% [10], exhibit somatic mutations in genes responsible for epigenetic regulation, such as *DNMT3A, TET2* and *ASXL1*, while only a minority have mutations in splicing genes like *SF3B1*, S*RSF2* and *U2AF1,* and *TP53,* whose prevalence range from 0.03 to 0.2% [11, 12].

The worldwide diffusion of next-generation sequencing (NGS) techniques has allowed the identification of somatic mutations at very low VAF in t-MN patients, emphasizing the concept of clonal evolution. However, characterizing the role of somatic mutations in t-MN patients remains challenging because of the limited sample sizes and the difficulties in collecting paired samples at primary diagnosis and t-MN, from patients who share the same primary tumor and treatment history.

In a recently-published paper, we investigated the prevalence of CHIP in a cohort of patients who developed a t-MN after treatment with chemo(immuno)therapy [13]. In the present study, we aim to expand our observations by conducting a review of data available in the literature in order to characterize the role of specific gene variants in t-MN development and to gain novel insights on malignant progression of individuals with CHIP exposed to cytotoxic treatments. Knowledge of the mechanisms underlying progression of CHIP will allow for better t-MN risk stratification and the development of patient-specific treatments.

## Patients and method

### Patient characteristics

**Fig. 1:**
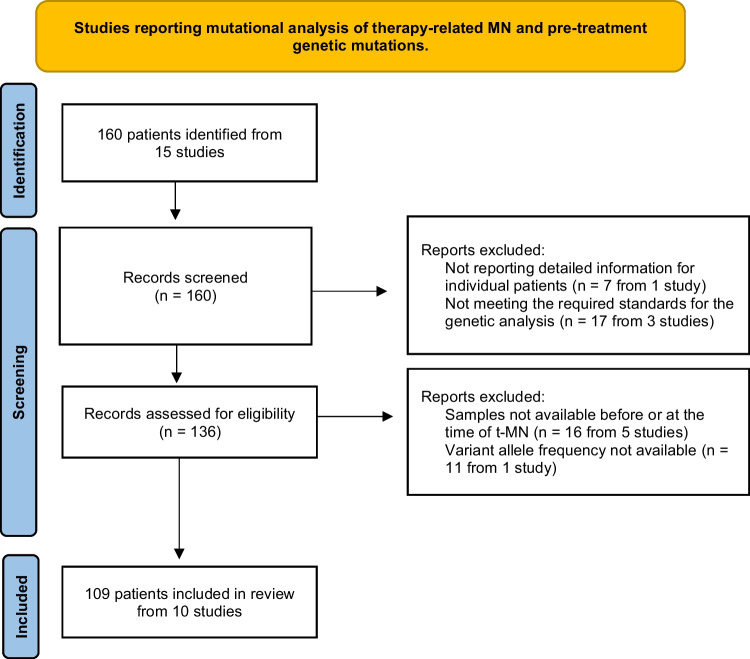
PRISMA flow diagram of patients enrollment

This systematic review was conducted according to the Preferred Reporting Items for Systematic Reviews and Meta-analyses (PRISMA) statements [14]. The literature search focused on studies reporting t-MNs with available detailed genetic information (including at least the 29 genes selected for the purpose of this study, see Supplementary Table 1) at the time of primary malignancy and at the time of t-MN. The systematic search retrieved 146 patients from 15 studies [6, 9, 13, 15–26]; after the eligibility check, 109 patients from 10 studies were selected [6, 13, 16, 17, 19–22, 24]. The PRISMA flow chart of the selection procedure and main reasons for exclusion are detailed in Fig. 1 (More details in supplementary material). Supplementary tables 2 and 3 show the studies included in the analysis, the gene panel selected for the analysis, the time of genetic screening and the variants identified. The nature of variants whose pathogenicity was not clearly stated in the original papers was assessed and clearly marked with asterisks (supplementary Table 3). We identified only 6 variants of uncertain significance (VUS), that for consistency with original papers were included in the analysis.

When available, the following data were collected: age at the time of primary tumor (t0), sex, type of primary malignancy, treatment received (including autologous stem cells transplant [ASCT]), type of t-MN, latency between primary malignancy and t-MN, genetic mutation at t0 and t-MN, source of genetic analysis at t0 and t-MN, karyotype analysis at t0 (none of the studies included presented this data) and t-MN (see Table 1). The patients were considered to have CHIP if the genetic mutations presented VAF ≥ 2%. To evaluate the mutational burden changes between t0 and t-MN, we identified a 10% cut-off to define a significant increased/decreased VAF. *TP53* mutations were considered multi-hit according to both WHO-5 and ICC classifications [3, 27]. Other mutations were considered to be multi-hit if either 2 different mutations on the same gene or a single mutation with VAF > 50%. Complex karyotype was defined by the presence of more than or equal to 3 aberrations.

### Statistical analysis

Univariate and multivariate analyses were used to establish associations between the variables. Categorical variables were compared using the chi-square test; odds ratios (OR) with 95% confidence intervals (CI:95%) were also calculated (unless one of the cells of the contingency table for the categorical variables is equal to 0). For expected cell values less than 5, Fisher’s exact test and the exact limits for confidence intervals were preferred. The Mann–Whitney test was used for continuous variables as appropriate. A p-value less than 0.05 was considered as statistically significant. Statistical analysis was performed through IBM SPSS Statistics 27 (IBM Corp. in Armonk, NY).

**Table 1:** Demographic data of patients enrolled. BM: bone marrow; F: female; M: male; NA: not available; PB: peripheral blood; t-MN: therapy-related myeloid neoplasm

Demographic and clinical data of 109 studied patients		
Age at primary tumor diagnosis	Years, median (Range) NA, n (%)	62 (18–79) 51 (47)
Gender	M, n (%) F, n (%) NA, n (%)	38 (35) 26 (24) 45 (41)
Primary tumour	Plasma cells dyscrasia, n (%) Lymphoid Malignancy, n (%) Solid Tumour, n (%)	18 (16) 63 (58) 28 (26)
Treatment	Chemotherapy, n (%) Chemoradiotherapy, n (%) Anthracycline- based treatment, n (%) Antimetabolites- based treatment, n (%) Immunomodulatory drugs- based treatment, n (%) NA, n (%) ASCT, n (%) NA for ASCT, n (%)	21 (19) 50 (46) 21 (19) 23 (21) 4 (4) 38 (35) 51 (47) 13 (12)
Mutations detected at the time of first malignancy	n, median (Range)	1 (0–4)
Source of analysis at the time of first malignancy	PB, n (%) BM, n (%)	101 (93) 8 (7)
Latency to t-MN development	months, median (Range)	36 (2–183)
Mutations detected at the time of t-MN	n, median (Range)	2 (0–7)
Source of analysis at the time of t-MN	BM, n (%) PB, n (%) NA, n (%)	68 (62) 21 (19) 20 (18)
Abnormal Karyotype at the time of t-MN	n (%) Complex Karyotype, n (%) del(7), n (%) del(5), n (%) NA, n (%)	54 (79) 28 (41) 13 (19) 12 (18) 41 (38)

## Results

### Demographic characteristics

One hundred and nine patients were included in this study (Median age 62 yr [range 18–79]; M/F ratio 1.5). The more frequent primary tumors were lymphoid malignancies (58%), solid tumors (26%) and plasma-cell dyscrasias (Multiple myeloma or AL amyloidosis, 16%). Data on previous therapies were available in 71 patients: 21 underwent chemotherapy and 50 a chemo-radiotherapy combination; ASCT was carried out in 47% of patients. The most common t-MN was MDS (61%), while AML was diagnosed in 38% of cases and the type of t-MN was not specified in 16% of patients. The median latency between t0 and t-MN was 36 months (range 2–183) (Table 1).

### Somatic lesions: from CHIP to myeloid malignancy

At the time of the primary malignancy, 76 gene mutations were identified in 47 patients (43%); 26 (24%) had 1 mutation, 14 (13%) had 2 mutations, 6 (5%) had 3 mutations and 1 (1%) had 4 mutations. At the time of t-MN, 210 gene mutations were identified in 92 patients (84%, p < 0.0001); 34 (31%) had 1 mutation, 27 (25%) had 2 mutations, 17 (16%) had 3 mutations, 5 (5%) had 4 mutations, 7 (6%) had 5 mutations, 1 (1%) had 6 mutations and 1 (1%) had 9 mutations (Supplementary Fig. 1).

**Fig. 2:**
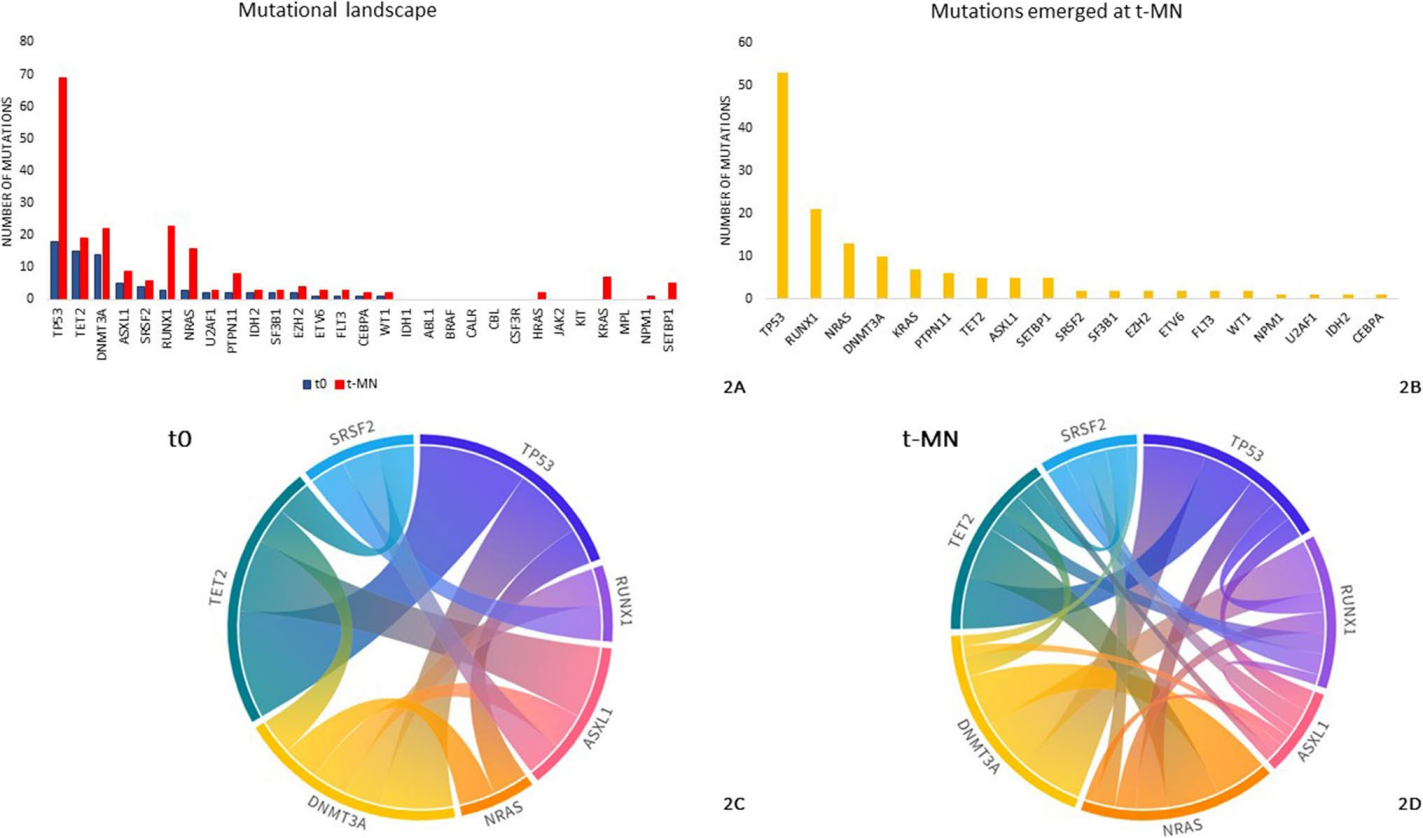
Mutational landscape in the patient population. **2A**) Number of mutations at the time of diagnosis of first malignancy (t0) and at t-MN development **2B** Landscape of mutations emerged at t-MN. **2C/D** Circle plots showing the co-mutation patterns in the genes most commonly mutated at t0 (**2C**) and at t-MN (**2D**)

The most common mutations at t0 were *TP53* (16%), *TET2* (12%), *DNMT3A* (12%) and *ASXL1* (4%) (Fig. 2). For all genes, there was an increase of VAF between t0 and t-MN, which was significant only for *TET2* (median VAF t0: 3.5% *vs* t-MN: 21.2%, p = 0.019) and *TP53* (median VAF t0: 5% *vs* t-MN: 31.95%, p = 0.005). Of note, in patients with *TP53* mutations at t0, both the INDEL mutations (n = 2) showed a VAF increasing over time, whereas missense mutations expanded in half of the cases (6 out of 12), but the difference did not reach statistical significance, probably due to the limited number of patients with INDEL mutations. In patients with *TP53*-driven CHIP with a stable VAF (variation ≤ 10%), the *TP53*^Mut^ clone at t-MN was not detectable in two cases (UPN 56 and 103 in supplementary Table 3). The first patient developed a t-MDS characterized by a single *GATA2* mutation (VAF: 33%), and inv(3) three years after treatment with Temozolomide and radiotherapy for Glioblastoma. The patient was treated with Fludarabine, Idarubicin and Cytarabine for the t-MN and progressed successfully to hematopoietic stem cell transplantation [6]. The second patient developed a t-MDS (subsequently evolved to t-AML) with complex karyotype, without genetic lesions after multiple chemo-radiotreatments for lymphoma [26]. No additional differences were detected between type of mutation and VAF changes (supplementary Fig. 2).

**Fig. 3:**
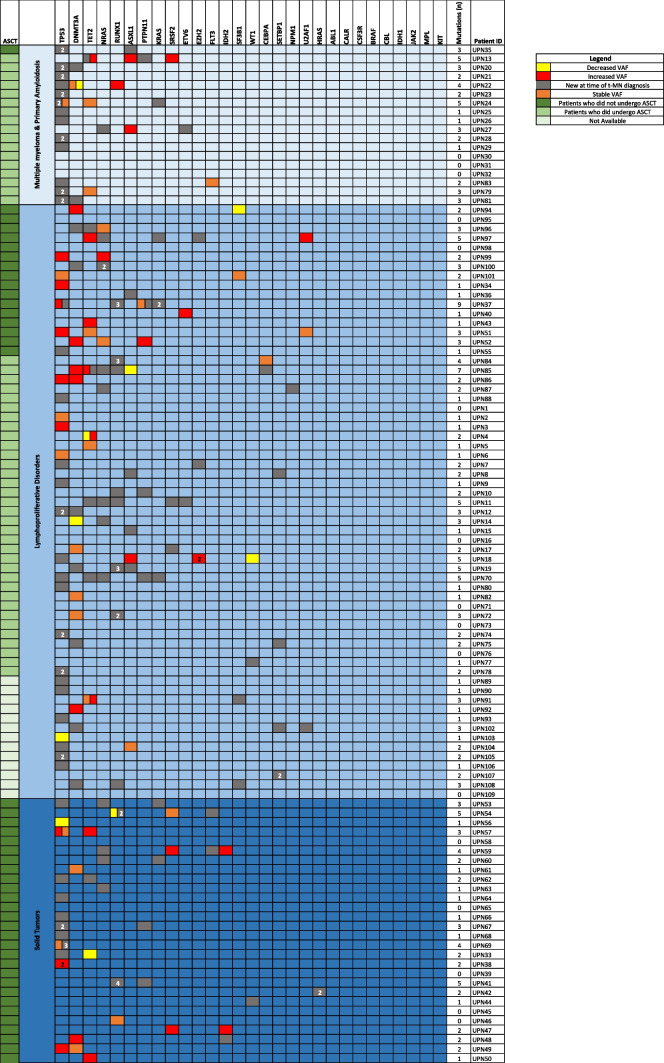
Mutational profile of the patients, grouped for type of primary tumor, with information on autologous stem cell transplant (ASCT). Each row refers to a single patient. The color of the box represents the changes in the VAF of the mutations between t0 and t-MN as indicated by the legend. NA: Not available. VAF: variant allele frequency

Most frequently mutated genes at t-MN were *TP53* (45%), *DNMT3A* (20%), *TET2* (15%) and *NRAS* (14%); all of these variants but *TET2* were more frequent when compared with t0 (p < 0.0001, p = 0.004, p = 0.375 and p = 0.003, respectively; Fig. 2). The most common mutations emerging at t-MN or not detectable at t0 were *TP53* (35%), *NRAS* (9%), *RUNX1* (9%) and *DNMT3A* (9%), although we cannot exclude that these mutations were present at values below the detection threshold declared by the study. In particular, *TP53* and *RUNX1* genes presented a high rate of multi-hit mutations (63% and 60%, respectively). A comprehensive representation of the dynamic mutational profile of the patients with information on primary tumor and ASCT is shown in Fig. 3.

**Fig. 4:**
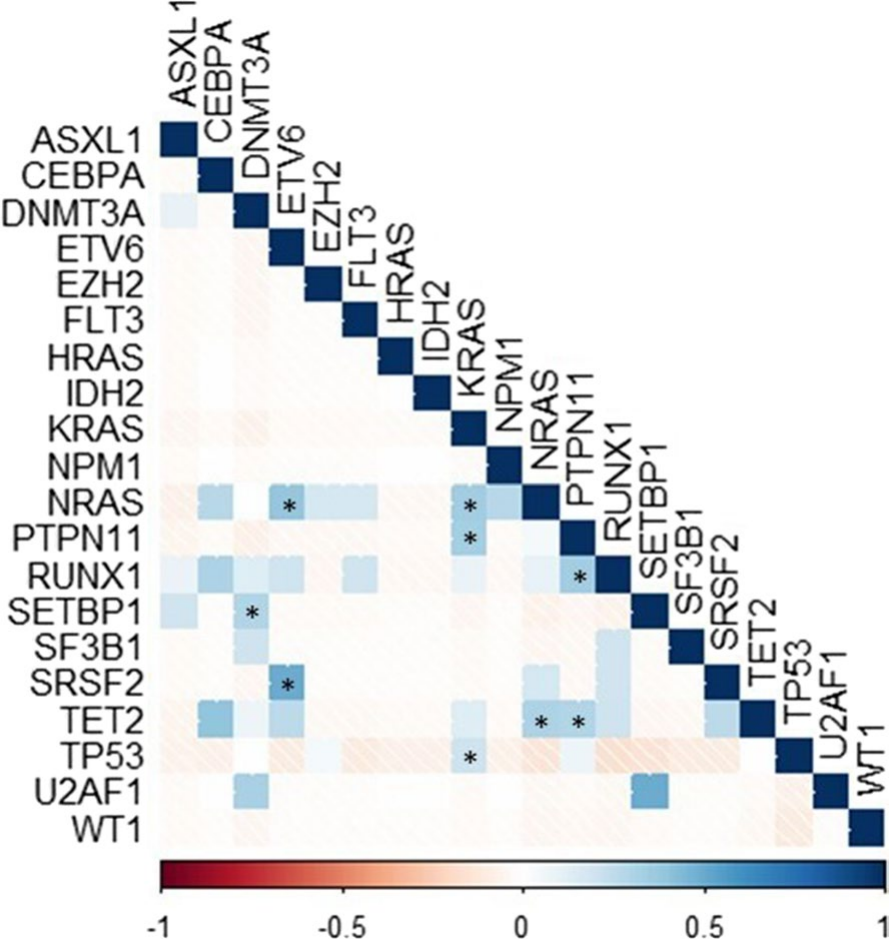
Correlation between mutations emerged at t-MN. The figure shows the correlations between mutations identified at t-MN but not present at t0. Asterisks indicate statistically significant correlations (p < 0.05). The strength of the correlation is indicated in color shades as shown by the legend below the figure. Only mutations occurring at least once at t-MN are shown. For further information please refer to supplementary Table 5

We then explored correlations between mutations at t0 and t-MN, with significant results summarized in Supplementary Tables 4–6. We identified several patterns of mutations co-occurring at t-MN and not detectable at t0, such as *ETV6-NRAS, ETV6-SRSF2, PTPN11-RUNX1, TET2-NRAS, KRAS-NRAS, PTPN11-TET2, KRAS-PTPN11, SETBP1-DNMT3A* and *TP53-KRAS* (Fig. 4). Furthermore, *ASXL1*-driven CHIP significantly correlated with the emergence of *TET2* and *CEBPA* mutations at t-MN (p = 0.024 OR 16.667, 95%CI: 2.147–209.981 and p = 0.046 OR NA, respectively), *U2AF1*-driven CHIP with *EZH2* (p = 0.037 OR 106.000 95%CI 3.537–3176.483), *IDH2* and *SRSF2*-driven CHIP with *FLT3* mutations (p = 0.037 OR 106.000 95%CI 3.537–3176.483 and p = 0.001 OR NA, respectively) and *DNMT3A*-driven CHIP with a lower incidence of *TP53* mutation (p = 0.031 OR 0.133 95%CI 0.017–1.065).

### Focus on main CHIP driver mutations

**Fig. 5:**
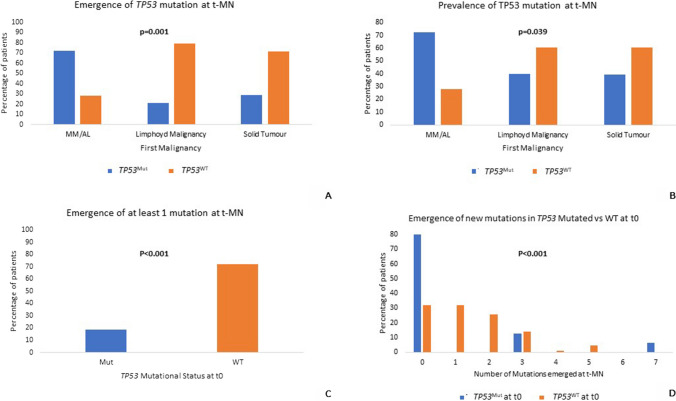
*TP53* mutation analysis. Correlations between the type of first malignancy and the presence of a newly-identified *TP53* mutation at t-MN (not detected at the type of fist malignancy) (**5A**) and the overall prevalence of *TP53* at t-MN (regardless of *TP53*-driven CHIP at the type of first malignancy) (**5B**). Correlations between *TP53* status at t0 and emergence of at least 1 mutation at t-MN (**5C**) and numbers of mutations emerged at t-MN (**5D**)

Given the well-known role of the *TP53* mutation in t-MN pathogenesis, we then investigated this mutation: patients who were *TP53*^*wt*^ at t0 developed no new mutations at t-MN diagnosis in 29% of cases, 1 new mutation in 29% of cases and 2 new mutations in 25% of cases. In contrast, patients who carring *TP53* mutation at t0 were more likely to have no new mutations at t-MN (13 patients, 81%), with a few developing a high number of new mutations (3 mutations in 2 patients, 12.5% and 7 mutations in 1 patient, 7%; Fig. 5). Overall, the presence of *TP53* mutation at t0 was associated with a lower incidence of new mutations at t-MN (p < 0.001, OR 0.094, 95%CI 0.025–0.358).

Furthermore, both the emergence of a new *TP53* mutation at t-MN and the presence of *TP53* mutation at t-MN correlated with a primary plasma-cell dyscrasia (p = 0.001 and p = 0.039, respectively; Fig. 5). Information about the treatment of patients with this type of primary tumor were available for only 5 patients. The only two patients undergoing treatment with Lenalidomide developed *TP53* mutations at t-MN not detected at t0 (in both cases the sensitivity reported for the NGS method was 1% VAF) [21].

As expected from previous reports, the other most frequently mutated genes at t0 were *TET2* (15 mutations in 13 patients), *DNMT3A* (14 mutation in 13 patients) and *ASXL1* (4 mutations in 4 patients).

We observed that patients with *ASXL1-*driven CHIP more likely acquired new mutations at t-MN (1 mutation in 2 patients, 40%, 2 mutations in 2 patients, 40% and 4 mutations in 1 patient, 20%, p < 0.001), but not those carrying *DNMT3A* and *TET2* mutation at t0 (Supplementary Fig. 3).

### Karyotype analysis

Karyotype analysis was available only at t-MN. Karyotype abnormalities were identified in 54 out of 68 patients (79%). Most common were complex karyotype (28 patients, 41%), del(7) (13 patients, 19%) and del(5) (12 patients, 18%). Correlations were detected between del(7) and *PTPN11* mutations at t0 (the only two patients with *PTPN11*-driven CHIP presented this karyotype abnormality, p = 0.034, OR NA), *TP53* mutations at t0 and complex karyotype (p = 0.001, OR 12.294 95%CI: 2.454–61.599) and *TP53* mutation at t-MN and complex karyotype (p < 0.001, OR 21.000 95%CI: 5.905–74.685) (Supplementary Fig. 4). Focusing on the subtype of t-MN with normal karyotype (14 patients, 20%), although not statistically significant, we found a different mutational landscape, enriched in *TET2*, *PTPN11*, *KRAS* and *NRAS* mutations (42%, 21%, 21% and 21%, respectively; see Supplementary Fig. 5). Of note, 71% of normal karyotype (NK) patients had at least one mutation at t0, while this frequency was lower in non-NK patients (42%). No significant differences were detected in number of mutations at t-MN or emergence of new mutations at t-MN.

## Discussion

The comparative analysis of genetic features of samples in patients at the time of first malignancy and at the time of developing t-MN allowed us to observe a significant higher frequency of genetic variants and cytogenetic abnormalities in the second time point, as expected, an increasing VAF of specific hits between t0 and t-MN, such as *TET2* and *TP53,* possibly indicating a main role in leukemogenesis, and a lower incidence of *TP53* mutation at t-MN in patients harbouring a *DNMT3A*-driven CHIP.

To gain insight into malignant progression of individuals harboring CHIP mutations undergoing cytotoxic treatments, we compared the genetic landscape of somatic mutations at t0 of the cohort of patients we gathered from literature to the one found in the non-hematological patients (NHP) as reported in the seminal manuscripts by Jaiswal et al*.* and Genovese et al*.* [12, 28]. The first difference that stands out is the higher rate of patients with CHIP in our cohort (43% vs 5% in age-matched individuals [60–65 yrs.]). It should be pointed out, though, that part of the samples at t0 were collected before t-MN onset but after some cycles of chemotherapy (e.g. at the time of hematopoietic stem cell [HSC] collection for ASCT), which could trigger a clone selection, and the possibility that mutations identified as CHIP drivers could have been found in clones responsible for the primary malignancies.

When compared to NHP, the patient cohort showed underrepresentation of *DNMT3A* (the most common mutation in NHP), *ASXL1* (whose mutation frequency in NHP is similar to *TET2*), *JAK2*, *SF3B1* and *CBL* mutations, with overrepresentation of *TP53* and *TET2* mutations. The importance in malignant progression of *TP53-* and *TET2*-driven CHIP is also confirmed by the significant VAF increase from t0 to t-MN.

As expected from previous reports, the cohort of t-MN selected for this study presented high rate of cytogenetic abnormalities, in particular del(5), del(7) and complex karyotype, and characteristic genetic signatures, with low incidence of mutations commonly detected in *de-novo* AML, such as *NPM1* and *FLT3*, and higher incidence of *TP53*, *NRAS* and *KRAS* mutations [4].

Recent reports pointed out that NK t-MN is an entity clinically and genetically distinct from classical t-MN harboring karyotype abnormalities. NK t-MN present better prognosis, lower frequency of *TP53* mutations and higher frequency of *TET2, NPM1, ASXL1, SRSF2, RUNX1, KRAS, FLT3* and *STAG2* mutations [29–31]. Our small cohort of NK t-MN is in line with some of the mutational features previously reported, as enrichment in *TET2* and *KRAS* mutations. Intriguingly, we detected a higher, although not statistically significant rate of CHIP at t0 when compared to non-NK t-MN (71% vs 42%). If confirmed in a larger cohort of patients, this difference could let us envision a different preferential mechanism of progression between the two entities: one CHIP-linked (through clone expansion and/or acquisition of additional mutations), more common in NK t-MN, *versus* correlated to chromosomal destabilization, specific for abnormal karyotype t-MN, characterized by *TP53* mutations at the time of first malignancy. The lack of cytogenetic analysis at t0 does not allow us to further speculate on the last mechanism, which could be fostered by karyotype abnormalities or single nucleotide polymorphisms before the onset of t-MN or be the consequence of the exposure to genotoxic agents over the *TP53*^mut^ karyotypic ground [4].

Current evidence on the mechanisms of progression of CHIP to MN highlight similarities and differences among specific gene-driven CHIPs. Among these, *DNMT3A, TET2* and *ASXL1* mutations are often detected at considerably higher VAF compared to *NRAS, KRAS* and *GNAS* [32, 33]. Analyzing a large cohort of elderly individuals, Van Zeventer et al., highlighted that *JAK2, TET2, ASXL1* and *TP53* somatic lesions, but not *DNMT3A*, tend to increase their allelic burden over time. Furthermore, *TET2* and *ASXL1*-mutated diseases present a higher propensity to acquire additional mutations over time, as compared to those carrying *DNMT3A* mutations [34]. Intriguingly, in the studied cohort, patients harboring *TET2* mutations at t0 did not show a different pattern of emerging mutations at t-MN, when compared to other patients; instead, *TET2* VAF significantly increased between t0 and t-MN. These findings lead to the hypothesis that *TET2*^mut^ clones could preferentially expand and not accumulate mutations, when exposed to cytotoxic therapy.

Several papers have focused on the leukemogenesis process of *TP53*^mut^ MN, recently recognized by ICC as an autonomous category due to the high-risk features and adverse outcomes [3, 35, 36]. In particular, *in-vitro* and *in-vivo* studies showed that cytotoxic therapy selects low-VAF *TP53*^mut^ clones which present survival advantage preferentially expanded after treatment [37]. In the same line, Bolton et al*.* documented a strong association between *TP53*-driven CH and previous exposure to cancer therapy [38]. Accordingly, Shah et al*.*, analyzing a cohort of nearly five hundred t-MN patients, found a high percentage of *TP53* mutations (37%). Strikingly, the total number of co-mutations was significantly inferior in *TP53*^mut^ cases compared to *TP53*^wt^, with more then 60% of *TP53*^mut^ patients not presenting co-mutations [39]. The small number of co-mutations detected by Shah et al*.* and confirmed in our literature review is consistent with the high rate of *TP53* biallelic mutations, feature found to worsen prognosis and linked to a smaller co-mutational burden when compared to monoallelic mutations [40, 41].

Taken together, this evidence highlights a discrepancy between the rate of cytogenetic abnormalities and the gene co-mutational burden in *TP53*^mut^ t-MN. Accordingly, several authors demonstrated the key role of TP53 in maintaining diploid karyotype [42–44]; more precisely, *TP53* induces apoptosis in cells that have a long pause in the mitotic checkpoint, which indicates DNA damage [45].

Taking advantage of CRISPR-Cas9 technology, Boettcher et al*.* demonstrated a dominant negative effect of *TP53* missense mutations, which would confer similar drug resistance and survival advantage when compared to *TP53*^ko^ models [46]. Intriguingly, in our patients gathered from the literature, the two patients carrying a truncating *TP53*-mutation at t0 showed increased VAF at t-MN, whereas among the 12 patients with a missense mutation, only half of them presented an increased VAF. Although not statistically significant, probably due to the low number of studied patients, this association could point out differences in therapy-related risk progression in different *TP53* mutations. Unfortunately, the small number of patients does not allow definitive conclusions and underlines the need to extend the study of the role of CHIP to larger cohorts of patients who develop t-MN following treatment with chemo- and/or radio-therapy.

Recently, Sperling et al. demonstrated, in in vitro and in vivo models, that lenalidomide, but not pomalidomide, provides a selective advantage to *TP53*^mut^ HSCs [47]. Similarly, in the present case series, we documented the emergence of *TP53* mutations in the two patients exposed to lenalidomide. Accordingly, it is worth highlighting that clonal hematopoiesis at a VAF below 2% (age-related clonal hematopoiesis, ARCH), was not included in our study, due to inconsistencies in the limit of resolution of selected papers, but could be more frequent than CHIP, as shown in NHP by previous reports [48, 49]. Thus, it is plausible to envision a larger rate of patients harboring clonal hematopoiesis at the time of primary malignancy; accordingly, several mutations emerging at t-MN (especially *TP53*) could be present also before the cytotoxic therapy and thus be selected thanks to the resistance to treatments.

In sum, the comparative analysis of molecular profiles pre- and post- t-MN onset allowed us to gain interesting insights on malignant progression in CHIP-carrying individuals, following exposure to cytotoxic treatments.

Our analysis of the data from both the perspective of CHIP-driven mutations and mutational landscape of t-MNs, corroborates the findings by previous authors and suggest a potential leukemogenic role of both *TET2* and *TP53*-driven CHIP. Although previous reports have showed an increasing of the *TET2* clones over time and in condition of aging/inflammation [34, 50], this is the first systematic analysis showing their potential role in the process of therapy-related leukemogenesis. Furthermore, we highlighted a preferential leukemogeneis process by *TP53*-driven CHIP, based on clone expansion rather than mutations acquisition. This feature resulted to be more common in clones harbouring INDEL *TP53* mutations, which, thus, could present an even higher risk of expansion when compared to missense mutations.

Intriguingly, patients carrying *DNMT3A* mutations at t0 presented a lower incidence of *TP53* variants at t-MN. This observation lines up with the lower trend to increase VAF over time and to acquire new mutations in *DNMT3A*-driven CHIP, both in patients developing t-MN and in healthy individuals, as shown by Van Zeventer et al. [34], and a lower risk of myeloid malignancy when compared to other genotypes [50]. In sum, these findings suggest that, when compared to other CHIP-drivers, *DNMT3A* presents lower leukemogenic potential and may eventually cause a milder disease phenotypes.

Unfortunately, the paucity of the data available did not allow further analyses to investigate specific therapy-related effects (e.g. ASCT or specific drug-based treatments) and to study more in deep gene-specific mutational patterns. In this regard, another limitation of our study is the exclusion of *PPM1D* from most gene panels, whose mutations are a known hematopoietic driver in response to DNA damage [51].

In this perspective, thanks also to the availability of biobanks in cancer centers, broader, comparative, prospective studies are warranted to further investigate CHIP progression mechanisms that could allow, in the near future, a better stratification of the individual risk of developing a t-MN. In this line, availability of alternative, and possibly targeted treatments, may better direct the therapeutic approach to hematologic malignancies and solid tumors, with the goal of reducing the rate of this unfavorable complication.

### Supplementary Information

Below is the link to the electronic supplementary material.

Supplementary file1 (DOCX 556 KB)

## Data Availability

The datasets generated and/or analyzed during the current study are available from the reports used for this review.
