# Supplementary material for: The role of clonal progression leading to the development of therapy-related myeloid neoplasms
Source: Ann Hematol. 2024 Jul 20;103(9):3507–17. doi: 10.1007/s00277-024-05803-y (PMC11358309; doi:10.1007/s00277-024-05803-y)
Supplement: Supplementary file 1 — Supplementary file1 (DOCX 556 KB) [file 277_2024_5803_MOESM1_ESM.docx]

**Supplementary Methods**

The literature search was carried out on the Pubmed platform including journals reporting ASH and EHA abstracts (Blood, HemaSphere). The search strategy included similar keywords: acute myeloid leukemia (AML), clonal hemopoiesis of indetermined potential (CHIP), myeloid neoplasm post cytotoxic therapy (MN-pCT), therapy related myeloid neoplasms (t-MN, including t-AML and t-MDS [Myelodysplastic syndrome]) and donor-derived leukemia. Two independent reviewers (LG and MRP) screened all of the titles and abstracts and evaluated each article. Disagreements were resolved by consensus and in concordance with a third reviewer (EF), when necessary. Studies that fulfilled the following criteria were included: studies reporting on myeloid malignancies arising after cytotoxic therapy with availability of detailed genetic information (including at least the 29 genes selected for the purpose of this study, see supplementary Table 1) at the time of primary malignancy and at the time of t-MN.

Part of the samples at t0 were collected before t-MN onset, but after some cycles of chemotherapy (e.g. at the time of hematopoietic stem cell [HSC] collection for ASCT). More details can be found in supplementary table 2.

**Supplementary Tables**

**Supplementary Table 1.** **Gene list considered in the analysis (n=29 genes, VAF cut-off: 2%).**

| ABL1 | FLT3 | PTPN11 |
| --- | --- | --- |
| ASXL1 | HRAS | RUNX1 |
| BRAF | IDH1 | SETBP1 |
| CALR | IDH2 | SF3B1 |
| CBL | JAK2 | SRSF2 |
| CEBPA | KIT | TET2 |
| CSF3R | KRAS | TP53 |
| DNMT3A | MPL | U2AF1 |
| ETV6 | NPM1 | WT1 |
| EZH2 | NRAS |  |

**Supplementary table 2. Characteristics of the studied included in the analysis.**

* The pathogenicity of the variants was not clearly stated in the papers.

CLL: chronic lymphocytic leukemia; NA: not available; WES: whole exome sequencing.

| **Authors** | **Year** | **Number of patients** | **Genetic screening methodology*** | **Cancer type included** | **Time of genetic screening at t0** |
| --- | --- | --- | --- | --- | --- |
| Gibson et al.^1^ | 2017 | 8 | WES | Lymphoma | Stem cell harvest |
| Xie et al.^2^ | 2023 | 8 | 298 genes panel | Lymphoma | Disease relapse |
| Voso et al.^3^ | 2022 | 8 | 54 genes panel | CLL | Pretreatment |
| Liu et al.^4^ | 2022 | 5 | 400 genes panel* | Lymphoma | NA |
| Gramegna et al.^5^ | 2022 | 19 | 45 genes panel* | Lymphoma/Multiple Myeloma | Stem cell harvest |
| Khalife-Hachem et al.^6^ | 2021 | 12 | 74 genes panel | Breast/Gynecologic | NA |
| Takahashi et al.^7^ | 2016 | 14 | 32 genes panel | Solid and hematological | Pretreatment |
| Gillis et al.^8^ | 2017 | 11 | 49 genes panel/WES | Solid and hematological | NA |
| Mouhieddine et al.^9^ | 2020 | 13 | 224 genes panel | Multiple Myeloma | Stem cell harvest |
| Soerensen et al.^10^ | 2022 | 11 | 30 genes panel* | Lymphoma/Multiple Myeloma | Stem cell harvest |

**Supplementary table 3. Variants identified at t0 and t-MN.**

In red the somatic hits emerged at tMN and not detected at to.

P= Pathogenic

LP= Likely pathogenic

VUS= variant of uncertain significance

* The pathogenicity of these variants was assessed because not clearly stated in original papers.

| **Reference** | **ID patient** | **Mutation t0** | **Position** | **P/LP/VUS** | **VAF (%)** | **Mutation tMN** | **Position** | **P/LP/VUS** | **VAF (%)** |  |
| --- | --- | --- | --- | --- | --- | --- | --- | --- | --- | --- |
| Gibson et al.^1^ | 1 |  |  |  |  |  |  |  |  |  |
|  | 2 | *TP53* | p.C275Y | P/LP | 2,6 | *TP53* | p.C275Y | P/LP | 2,1 |  |
|  | 3 | *TP53* | p.C182delinsWX | P/LP | 12,1 | *TP53* | p.C182delinsWX | P/LP | 26 |  |
|  | 4 | *TET2* | p.I1873T | P/LP | 32 | *TET2* | p.I1873T | P/LP | 45,3 |  |
|  |  |  | p.R544X | P/LP | 3,2 |  |  |  |  |  |
|  | 5 | *TET2* | p.T1895fs | P/LP | 4,1 | *TET2* | p.T1895fs | P/LP | 10,1 |  |
|  | 6 | *TP53* | p.V173M | P/LP | 4,2 | *TP53* | p.V173M | P/LP | 6,4 |  |
|  | 7 |  |  |  |  | *EZH2* | p.Y602N | P/LP | 3,7 |  |
|  |  |  |  |  |  | *TP53* | p.R282W | P/LP | 9,5 |  |
|  | 8 |  |  |  |  | *ASXL1* | p.G642fs | P/LP | 29,1 |  |
|  |  |  |  |  |  | *SETBP1* | p.D868N | P/LP | 73,8 |  |
| Soerensen et al.^10^ | 9* |  |  |  |  | *TP53* | p.R248Q | P/LP | 65,8 |  |
|  | 10* |  |  |  |  | *PTPN11* | p.S502L | P/LP | 22,8 |  |
|  |  |  |  |  |  | *RUNX1* | p.A142Lfs*3 | P/LP | 26,2 |  |
|  | 11* |  |  |  |  | *ETV6* | p.L97P | VUS | 4,7 |  |
|  |  |  |  |  |  | *NRAS* | p.G12D | P/LP | 17,3 |  |
|  |  |  |  |  |  | *RUNX1* | p.D198N | P/LP | 56,6 |  |
|  |  |  |  |  |  | *SRSF2* | p.P95H | P/LP | 45,7 |  |
|  |  |  |  |  |  | *TET2* | p.Q1030Sfs* | P/LP | 57,9 |  |
|  | 12* |  |  |  |  | *DNMT3A* | c.1015-1G>C (p?) | P/LP | 2,3 |  |
|  |  |  |  |  |  | *TP53* | p.Y220C | P/LP | 25,6 |  |
|  |  |  |  |  |  |  | p.N131T | P/LP | 25,8 |  |
|  | 13* | *ASXL1* | p.G646Wfs*12 | P/LP | 30,8 | *ASXL1* | p.G646Wfs*12 | P/LP | 45,5 |  |
|  |  | *SRSF2* | p.P95H | P/LP | 34,8 | *SRSF2* | p.P95H | P/LP | 47,2 |  |
|  |  | *TET2* | p.V1417Sfs*9 | P/LP | 2,7 | *TET2* | p.V1417Sfs*9 | P/LP | 15,2 |  |
|  |  |  |  |  |  | *PTPN11* | p.E69K | P/LP | 17,6 |  |
|  |  |  |  |  |  | *TET2* | c.4044+1G>A (p?) | P/LP | 15,4 |  |
|  | 14* | *DNMT3A* | c.1474+1G>A | P/LP | 20,3 | *DNMT3A* | c.1474+1G>A (p?) | P/LP | 2,1 |  |
|  |  |  |  |  |  | *NRAS* | p.G13R | P/LP | 44,1 |  |
|  | 15* |  |  |  |  | *ASXL1* | p.Q512* | P/LP | 31 |  |
|  | 16* |  |  |  |  |  |  |  |  |  |
|  | 17* | *DNMT3A* | p.R604Q | P/LP | 47,5 | *DNMT3A* | p.R604Q | VUS | 48,3 |  |
|  |  |  |  |  |  | *SRSF2* | p.P95R | P/LP | 43,2 |  |
|  | 18* | *ASXL1* | p.S689* | P/LP | 12,1 | *ASXL1* | p.S689* | P/LP | 49,4 |  |
|  |  | *EZH2* | p.I375Rfs*50 | P/LP | 10,8 | *EZH2* | p.I375Rfs*50 | P/LP | 49,3 |  |
|  |  |  | p.R566C | P/LP | 11,8 |  | p.R566 | P/LP | 50,3 |  |
|  |  | *WT1* | p.R434_S435insTH | P/LP | 9,5 | *TP53* | p.R273H | P/LP | 99,2 |  |
|  | 19* |  |  |  |  | *ASXL1* | p.G646Wfs*12 | P/LP | 43,6 |  |
|  |  |  |  |  |  | *DNMT3A* | p.S663* | P/LP | 49,2 |  |
|  |  |  |  |  |  | *RUNX1* | p.G165R | P/LP | 11,6 |  |
|  |  |  |  |  |  |  | p.R204* | P/LP | 31,3 |  |
|  |  |  |  |  |  |  | p.A338Rfs*? | P/LP | 4 |  |
| Mouhieddine et al.^9^ | 20 |  |  |  |  | *TP53* | p.C141Y | P/LP | 45,8 |  |
|  |  |  |  |  |  |  | p.M237I | P/LP | 40 |  |
|  |  |  |  |  |  | *DNMT3A* | Splice (25464578: T, C) | P/LP | 45,7 |  |
|  | 21 |  |  |  |  | *TP53* | p.L194H | P/LP | 19,2 |  |
|  |  |  |  |  |  |  | p.N131T | P/LP | 5,9 |  |
|  | 22 | *DNMT3A* | Splice (25469919: C, T) | P/LP | 4,9 | *DNMT3A* | Splice (25469919: C, T) | P/LP | 20,2 |  |
|  |  |  | p.F909S | P/LP | 2,4 | *RUNX1* | p.R135K | P/LP | 22,4 |  |
|  |  | *RUNX1* | p.R135K | P/LP | 4,7 | *TP53* | p.R248W | P/LP | 6,1 |  |
|  | 23 |  |  |  |  | *TP53* | Splice (7574035: T, A) | P/LP | 32,3 |  |
|  |  |  |  |  |  |  | p.Y220C | P/LP | 26 |  |
|  | 24 | *TP53* | p.R248W | P/LP | 4 | *TP53* | p.R248W | P/LP | 5,8 |  |
|  |  | *TET2* | p.QKPEQL317fs | P/LP | 14,2 | *TP53* | p.R282G | P/LP | 26,9 |  |
|  |  |  |  |  |  |  | Splice (7578291: T, G) | P/LP | 27,8 |  |
|  |  |  |  |  |  | *TET2* | p.QKPEQL317fs | P/LP | 21,3 |  |
|  |  |  |  |  |  | *KRAS* | p.G13R | P/LP | 5,6 |  |
|  | 25 |  |  |  |  | *TP53* | p.K164E | P/LP | 23,8 |  |
|  | 26 |  |  |  |  | *TP53* | p.E258K | P/LP | 14,6 |  |
|  | 27 | *ASXL1* | p.Q858* | P/LP | 4,5 | *ASXL1* | p.Q858* | P/LP | 46 |  |
|  |  |  |  |  |  | *ETV6* | p.D372G | P/LP | 63,9 |  |
|  |  |  |  |  |  | *NRAS* | p.Q61L | P/LP | 7,4 |  |
|  | 28 |  |  |  |  | *TP53* | p.R306* | P/LP | 43,3 |  |
|  |  |  |  |  |  |  | Splice (7577498: C, T) | P/LP | 44,1 |  |
|  | 29 |  |  |  |  | *TP53* | p.A159V | P/LP | 50 |  |
|  | 30 |  |  |  |  |  |  |  |  |  |
|  | 31 |  |  |  |  |  |  |  |  |  |
|  | 32 |  |  |  |  |  |  |  |  |  |
| Gillis et al.^8^ | 33 | *TET2* | frameshift, deletion | P/LP | 28,5 | *TET2* | frameshift, deletion | P/LP | 7,5 |  |
|  |  |  |  |  |  | *TP53* | nonsynonymous SNV | P/LP | 84,1 |  |
|  | 34 | *TP53* | frameshift deletion | P/LP | 14,6 | *TP53* | frameshift deletion | P/LP | 53,1 |  |
|  | 35 |  |  |  |  | *ASXL1* | nonsynonymous SNV | P/LP | 32,9 |  |
|  |  |  |  |  |  | *TP53* | frameshift | P/LP | 40,5 |  |
|  |  |  |  |  |  |  | nonsynonymous SNV | P/LP | 39,8 |  |
|  | 36 |  |  |  |  | *ASXL1* | nonframeshift substitution | P/LP | 4,6 |  |
|  | 37 | *PTPN11* | nonsynonymous SNV | P/LP | 3 | *KRAS* | nonsynonymous SNV | P/LP | 19,5 |  |
|  |  | *TP53* | nonsynonymous SNV | P/LP | 4,8 |  | nonsynonymous SNV | P/LP | 19,7 |  |
|  |  |  |  |  |  | *PTPN11* | nonsynonymous SNV | P/LP | 12,3 |  |
|  |  |  |  |  |  |  | nonsynonymous SNV | P/LP | 12 |  |
|  |  |  |  |  |  | *RUNX1* | nonframeshift insertion | P/LP | 22 |  |
|  |  |  |  |  |  |  | nonframeshift insertion | P/LP | 46 |  |
|  |  |  |  |  |  |  | nonsynonymous SNV | P/LP | 3,8 |  |
|  |  |  |  |  |  | *TP53* | nonsynonymous SNV | P/LP | 48,8 |  |
|  |  |  |  |  |  |  | nonsynonymous SNV | P/LP | 46 |  |
|  | 38 | *TP53* | nonsynonymous SNV | P/LP | 3,7 | *TP53* | nonsynonymous SNV | P/LP | 38 |  |
|  |  |  | nonsynonymous SNV | P/LP | 3,9 |  | nonsynonymous SNV | P/LP | 40 |  |
|  | 39 |  |  |  |  |  |  |  |  |  |
|  | 40 | *ETV6* | nonframeshift substitution | P/LP | 2,5 | *ETV6* | nonframeshift substitution | P/LP | 35,7 |  |
|  | 41 |  |  |  |  | *PTPN11* | nonsynonymous SNV | P/LP | 10,7 |  |
|  |  |  |  |  |  | *RUNX1* | frameshift insertion | P/LP | 20,2 |  |
|  |  |  |  |  |  |  | frameshift substitution | P/LP | 16,4 |  |
|  |  |  |  |  |  |  | frameshift insertion | P/LP | 11,7 |  |
|  |  |  |  |  |  |  | frameshift substitution | P/LP | 22,3 |  |
|  | 42 |  |  |  |  | *HRAS* | nonsynonymous SNV | P/LP | 3,6 |  |
|  |  |  |  |  |  |  | nonsynonymous SNV | P/LP | 3,2 |  |
|  | 43 | *TET2* | Stopgain | P/LP | 3,3 | *TET2* | Stopgain | P/LP | 27 |  |
| Takahashi et al.^7^ | 44 |  |  |  |  | *WT1* | p.S381X | P/LP | 14,7 |  |
|  | 45 |  |  |  |  |  |  |  |  |  |
|  | 46 | *RUNX1* | p.L98fs | P/LP | 3,7 | *RUNX1* | p.L98fs | P/LP | 23,3 |  |
|  | 47 | *IDH2* | p.R140Q | P/LP | 15,8 | *IDH2* | p.R140Q | P/LP | 45,5 |  |
|  |  | *SRSF2* | p.P95delinsRP |  | 13,7 | *SRSF2* | p.P95delinsRP | P/LP | 25,3 |  |
|  | 48 | *DNMT3A* | p.R882P | P/LP | 19,9 | *DNMT3A* | p.R882P | P/LP | 33,1 |  |
|  |  |  |  |  |  | *IDH2* | p.R172K | P/LP | 15,5 |  |
|  | 49 | *DNMT3A* | c.1475-2A>C | P/LP | 3,6 | *DNMT3A* | c.1475-2A>C | P/LP | 2,5 |  |
|  |  | *TP53* | p.H193R | P/LP | 22,3 | *TP53* | p.H193R | P/LP | 73,1 |  |
|  | 50 | *TET2* | p.L1212X | P/LP | 5,3 | *TET2* | p.L1212X | P/LP | 45,3 |  |
|  | 51 | *TET2* | p.Y1255X | P/LP | 8,4 | *TET2* | p.Y1255X | P/LP | 18,1 |  |
|  |  | *TP53* | p.Y205C | P/LP | 8,6 | *TP53* | p.Y205C | P/LP | 22,3 |  |
|  |  | *U2AF1* | p.Q157P | P/LP | 4 | *U2AF1* | p.Q157P | P/LP | 11,7 |  |
|  | 52 | *DNMT3A* | p.R882C | P/LP | 18,8 | *DNMT3A* | p.R882C | P/LP | 47,1 |  |
|  |  | *NRAS* | p.G13V | P/LP | 7,6 | *NRAS* | p.G13V | P/LP | 8,8 |  |
|  |  | *PTPN11* | p.G60V | P/LP | 4,3 | *PTPN11* | p.G60V | P/LP | 14,8 |  |
|  | 53 |  |  |  |  | *KRAS* | p.G12A | P/LP | 10,3 |  |
|  |  |  |  |  |  | *NRAS* | p.G13R | P/LP | 17,1 |  |
|  |  |  |  |  |  | *TP53* | p.Y107X | P/LP | 97,2 |  |
|  | 54 | *RUNX1* | c.509/1G>T | P/LP | 2,6 | *FLT3* | p.D593delinsEAPGEVD | P/LP | 22,4 |  |
|  |  | *SRSF2* | p.P95H | P/LP | 37 | *RUNX1* | p.R204X | P/LP | 30,7 |  |
|  |  |  |  |  |  |  | p.G165fs | P/LP | 20,7 |  |
|  |  |  |  |  |  | *SRSF2* | p.P95H | P/LP | 33,6 |  |
|  | 55 |  |  |  |  | *TP53* | p.L194H | P/LP | 42 |  |
|  | 56 | *TP53* | p.H193R | P/LP | 2,3 |  |  |  |  |  |
|  | 57 | *TP53* | p.R156H | P/LP | 54,1 | *TP53* | p.R156H | P/LP | 58,6 |  |
|  |  |  | p.R267Q | P/LP | 42,1 |  | p.R267Q | P/LP | 52,5 |  |
|  |  | *TET2* | p.H1380Y | P/LP | 8,7 | *TET2* | p.H1380Y | P/LP | 21,2 |  |
| Khalife-Hachem et al.^6^ | 58 |  |  |  |  |  |  |  |  |  |
|  | 59 | *IDH2* | p.R140L | P/LP | 7 | *IDH2* | p.R140L | P/LP | 47 |  |
|  |  | *SRSF2* | p.P95R | P/LP | 7,2 | *SRSF2* | p.P95R | P/LP | 47 |  |
|  |  |  |  |  |  | *FLT3* | p.V592A | P/LP | 2 |  |
|  |  |  |  |  |  | *NRAS* | p.G12D | P/LP | 10 |  |
|  |  |  |  |  |  | *ASXL1* | p.G643_G644fs | P/LP | 18 |  |
|  | 60 |  |  |  |  | *KRAS* | p.G12D | P/LP | 3 |  |
|  |  |  |  |  |  | *NRAS* | p.G12C | P/LP | 37 |  |
|  | 61 | *DNMT3A* | p.R729W | P/LP | 9 | *DNMT3A* | p.R729W | P/LP | 9 |  |
|  | 62 |  |  |  |  | *TET2* | p.Q383* | P/LP | 74 |  |
|  |  |  |  |  |  | *TP53* | p.R248W | P/LP | 64 |  |
|  | 63 |  |  |  |  | *NRAS* | p.G12D | P/LP | 3 |  |
|  | 64 |  |  |  |  | *TP53* | p.Sls347T | P/LP | 8 |  |
|  | 65 |  |  |  |  |  |  |  |  |  |
|  | 66 |  |  |  |  | *TP53* | p.C242Y | P/LP | 37 |  |
|  | 67 |  |  |  |  | *PTPN11* | p.G483A | P/LP | 2 |  |
|  |  |  |  |  |  | *TP53* | splice donor +1 | P/LP | 16 |  |
|  |  |  |  |  |  |  | c.933+1G>C p.Q52* | P/LP | 40 |  |
|  | 68 |  |  |  |  | *TP53* | p.K132R | P/LP | 87 |  |
|  | 69 | *TP53* | p.C176F | P/LP | 2,8 | *TP53* | p.C176F | P/LP | 3 |  |
|  |  |  |  |  |  | *TP53* | p.C141G | P/LP | 11 |  |
|  |  |  |  |  |  |  | p.K164E | P/LP | 2 |  |
|  |  |  |  |  |  |  | p.V143G | P/LP | 3 |  |
| Gramegna et al.^5^ | 70* |  |  |  |  | *KRAS* | p.Q61H | P/LP | 6,5 |  |
|  |  |  |  |  |  | *NRAS* | p.G12D | P/LP | 4,8 |  |
|  |  |  |  |  |  | *PTPN11* | p.A72T | P/LP | 3,5 |  |
|  |  |  |  |  |  | *TET2* | p.Q70Rfs*5 | P/LP | 34,2 |  |
|  |  |  |  |  |  | *TP53* | p.L145P | P/LP | 73,6 |  |
|  | 71* |  |  |  |  |  |  |  |  |  |
|  | 72* | *DNMT3A* | p.R882H | P/LP | 43 | *DNMT3A* | p.R882H | P/LP | 39,5 |  |
|  |  |  |  |  |  | *RUNX1* | p.D198N | P/LP | 2,6 |  |
|  |  |  |  |  |  |  | p.T358Dfs*242 | P/LP | 32,2 |  |
|  | 73* |  |  |  |  |  |  |  |  |  |
|  | 74* |  |  |  |  | *TP53* | p.Y234X | P/LP | 23,7 |  |
|  |  |  |  |  |  |  | p.C135Y | P/LP | 22 |  |
|  | 75* |  |  |  |  | *DNMT3A* | p.L798Ffs*4 | P/LP | 18 |  |
|  |  |  |  |  |  | *SETBP1* | p.D868N | P/LP | 17,1 |  |
|  | 76* |  |  |  |  |  |  |  |  |  |
|  | 77* |  |  |  |  | *WT1* | p.A204Cfs*49 | P/LP | 11,3 |  |
|  | 78* |  |  |  |  | *TP53* | p.P278S | P/LP | 13,2 |  |
|  |  |  |  |  |  |  | p.C141Y | P/LP | 10,4 |  |
|  | 79* | *TET2* | p.I1177S | P/LP | 2,8 | *TP53* | p.Y220C | P/LP | 31,7 |  |
|  |  |  |  |  |  |  | p.R110G | P/LP | 36,4 |  |
|  | 80* |  |  |  |  | *TP53* | p.H179R | P/LP | 78,9 |  |
|  | 81* |  |  |  |  | *DNMT3A* | Splice_donor_+1 (p?) | VUS | 39,5 |  |
|  |  |  |  |  |  | *TP53* | p.P278S | P/LP | 51,8 |  |
|  |  |  |  |  |  |  | p.R337C | P/LP | 43,4 |  |
|  |  |  |  |  |  |  |  |  |  |  |
|  | 82* | *DNMT3A* | p.Y735S | P/LP | 5,8 | *DNMT3A* | p.Y735S | P/LP | 4,1 |  |
|  | 83* | *FLT3* | p.A680V | P/LP | 43 | *FLT3* | p.A680V | P/LP | 46,5 |  |
|  |  |  |  |  |  | *TP53* | p.V143M | P/LP | 77,5 |  |
|  | 84* | *CEBPA* | p.H195_P196dup VUS | VUS | 12,4 | *CEBPA* | p.H195_P196dup | VUS | 11,2 |  |
|  |  |  |  |  |  | *RUNX1* | p.R169S | P/LP | 5,3 |  |
|  |  |  |  |  |  |  | p.S369Yfs*22 | P/LP | 11,6 |  |
|  |  |  |  |  |  |  | p.H404Pfs*196 | P/LP | 23 |  |
|  | 85* | *DNMT3A* | p.R882H | P/LP | 21,2 | *DNMT3A* | p.R882H | P/LP | 95 |  |
|  |  | *TET2* | p.G805X | P/LP | 48 | *TET2* | p.G805X | P/LP | 44,6 |  |
|  |  | *ASXL1* | p.W796Gfs*22 | P/LP | 2,7 | *CEBPA* | p.S348Rfs*? | P/LP | 36,5 |  |
|  |  |  |  |  |  | *NRAS* | p.Q61K | P/LP | 40 |  |
|  |  |  |  |  |  | *RUNX1* | p.R346Pfs*? | P/LP | 30,4 |  |
|  |  |  |  |  |  | *TET2* | p.G1275E VUS | VUS | 40,3 |  |
|  | 86* | *DNMT3A* | p.R882H | P/LP | 22.1. | *DNMT3A* | p.R882H | P/LP | 38,2 |  |
|  |  | *TP53* | p.R175H | P/LP | 13,8 | *TP53* | p.R175H | P/LP | 49,2 |  |
|  | 87* |  |  |  |  | *NPM1* | p.W288Cfs*? | P/LP | 30,4 |  |
|  |  |  |  |  |  | *NRAS* | p.G12D | P/LP | 44,7 |  |
|  | 88* |  |  |  |  | *TP53* | p.R175H | P/LP | 39,2 |  |
| Liu et al.^4^ | 89* |  |  |  |  | *TP53* | p.R248Q | P/LP | 97 |  |
|  | 90* |  |  |  |  | *TP53* | p.H179D | P/LP | 35 |  |
|  | 91* | *TET2* | p.K1094* | P/LP | 2 | *TET2* | p.K1094* | P/LP | 3 |  |
|  |  | *TET2* | p.S1284F | P/LP | 2 |  | p.S1284F | VUS | 31 |  |
|  |  |  |  |  |  | *SF3B1* | p.K666N | P/LP | 3 |  |
|  | 92* | *DNMT3A* | p.R326C | P/LP | 2 | *DNMT3A* | p.R326C | P/LP | 46 |  |
|  | 93* |  |  |  |  | *TP53* | p.C141Y | P/LP | 3 |  |
| Voso et al.^3^ | 94 | *DNMT3A* | p.R882H | P/LP | 2,3 | *DNMT3A* | p.R882H | P/LP | 40 |  |
|  |  | *SF3B1* | p.K700E | P/LP | 36 |  |  |  |  |  |
|  | 95 |  |  |  |  |  |  |  |  |  |
|  | 96 | *NRAS* | p.G13D | P/LP | 21 | *NRAS* | p.G13D | P/LP | 17 |  |
|  |  |  |  |  |  | *DNMT3A* | p.Y735C | P/LP | 3 |  |
|  |  |  |  |  |  | *TET2* | p.K1310fs*1 | P/LP | 2 |  |
|  | 97 | *TET2* | p.Q1445* | P/LP | 4 | *TET2* | p.Q1445* | P/LP | 95 |  |
|  |  | *U2AF1* | p.Q157R | P/LP | 6 | *U2AF1* | p.Q157R | P/LP | 45 |  |
|  |  |  |  |  |  | *EZH2* | p.R690H | P/LP | 6 |  |
|  |  |  |  |  |  | *KRAS* | p.T58I | P/LP | 21 |  |
|  |  |  |  |  |  | *NRAS* | p.G12S | P/LP | 3 |  |
|  | 98 |  |  |  |  |  |  |  |  |  |
|  | 99 | *NRAS* | p.G13R | P/LP | 10,1 | *NRAS* | p.G13R | P/LP | 38 |  |
|  |  | *TP53* | p.C242Y | P/LP | 21 | *TP53* | p.C242Y | P/LP | 92 |  |
|  | 100 |  |  |  |  | *DNMT3A* | c.1097_1098+3delAGGTA | P/LP | 44 |  |
|  |  |  |  |  |  | *NRAS* | p.G12S | P/LP | 12 |  |
|  |  |  |  |  |  |  | p.Q61K | P/LP | 52 |  |
|  | 101 | *SF3B1* | p.G742D | P/LP | 4,7 | *SF3B1* | p.G742D | P/LP | 12 |  |
|  |  | *TP53* | p.R209K | P/LP | 5,1 | *TP53* | p.R209K | P/LP | 9 |  |
| Xie et al.^2^ | 102 |  |  |  |  | *DNMT3A* | NA | P/LP | 46 |  |
|  |  |  |  |  |  | *SETBP1* | NA | P/LP | 45 |  |
|  |  |  |  |  |  | *U2AF1* | NA | P/LP | 15 |  |
|  | 103 | *TP53* | NA |  | 5 |  |  |  |  |  |
|  | 104 | *ASXL1* | NA |  | 3 | *TP53* | NA | P/LP | 89 |  |
|  | 105 |  |  |  |  | *TP53* | NA | P/LP | 3,6 |  |
|  |  |  |  |  |  |  | NA | P/LP | 3,7 |  |
|  | 106 |  |  |  |  | *TP53* | NA | P/LP | 60 |  |
|  | 107 |  |  |  |  | *SETBP1* | NA | P/LP | 17,8 |  |
|  |  |  |  |  |  |  | NA | P/LP | 7,8 |  |
|  | 108 |  |  |  |  | *DNMT3A* | NA | P/LP | 40 |  |
|  |  |  |  |  |  | *RUNX1* | NA | P/LP | 8 |  |
|  |  |  |  |  |  | *SF3B1* | NA | P/LP | 41 |  |
|  | 109 |  |  |  |  |  |  |  |  |  |

**Supplementary Table 4. Significant correlations between mutation type at t0 and at t-MN onset.**

| Correlations between mutations present at t0 | | |
| --- | --- | --- |
| Gene Mutations | **p** | **OR, 95% C.I.** |
| *IDH2-SRSF2* | 0.001 | NA |
| *U2AF1-TET2* | 0.015 | NA |
| *ASXL1-WT1* | 0.046 | NA |
| Correlations between mutations present at t-MN | | |
| Gene Mutations | **p** | **OR, 95% C.I.** |
| *TP53-SRSF2* | 0.032 | NA |
| *TET2-KRAS* | 0.040 | 6.923, 1.261-37.999 |
| *SRSF2-FLT3* | 0.007 | 51.000, 3.788-686.679 |
| *RUNX1-CEBPA* | 0.011 | NA |
| *RUNX1-PTPN11* | 0.028 | 7.750, 1.495-40.189 |
| *NRAS-KRAS* | 0.002 | 18.600, 3.019-114.612 |
| *KRAS-PTPN11* | 0.047 | 9.8, 1.436-66.883 |
| *NRAS-ETV6* | 0.043 | 15.667, 1.319-186.055 |

**Supplementary Table 5. Significant correlations between mutations emerged at t-MN.**

| Correlations between mutations emerged at t-MN | | |
| --- | --- | --- |
| Gene Mutations | **p** | **OR, 95% C.I.** |
| *NRAS-ETV6* | 0.011 | NA |
| *SRSF2-ETV6* | 0.037 | 106.000, 3.537-3176,483 |
| *PTPN11-RUNX1* | 0.010 | 13.714, 2.325-80.899 |
| *TET2-NRAS* | 0.017 | 10.444, 1.833-59.524 |
| *KRAS-NRAS* | 0.009 | 15.833, 2.332-107.495 |
| *TET2-PTPN11* | 0.035 | 12.375, 1.726-88.723 |
| *KRAS-PTPN11* | 0.027 | 15.333, 1.974-119.132 |
| *SETBP1-DNMT3A* | 0.034 | 14.000, 1.706-114.856 |
| *TP53-KRAS* | 0.049 | 8.235, 0.886-76.532 |

**Supplementary Table 6. Significant correlations between mutations present at t0 and emerging at t-MN**

| Correlations between mutations at t0 and emergence of mutations at t-MN | | |
| --- | --- | --- |
| Gene Mutations | **p** | **OR, 95% C.I.** |
| *ASXL1 t0-TET2 t-MN* | p= 0.024 | 16.667, 2.147-209.981 |
| *ASXL1 t0-CEBPA t-MN* | p= 0.046 | NA |
| *DNMT3A t0–TP53 t-MN* | p= 0.031 | 0.133, 0.017 -1.065 |
| *U2AF1 t0–EZH2 t-MN* | p= 0.037 | 106.000, 3.537-3176.483 |
| *IDH2 t0–FLT3 t-MN* | p= 0.037 | 106.000, 3.537-3176.483 |
| *SRSF2 t0–FLT3 t-MN* | p= 0.001 | NA |

**Supplementary Figures**

**Supplementary Figure 1. Number of mutations per patient, at t0 (A) and at t-MN onset (B).**


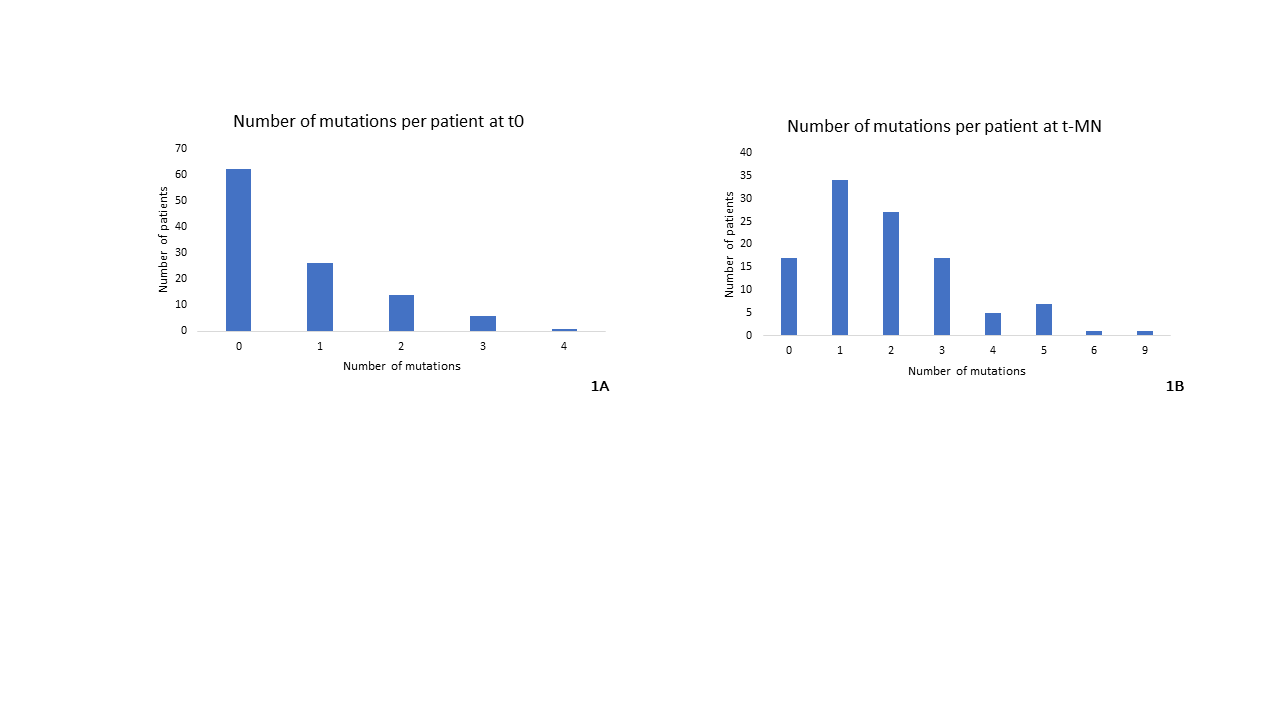


**Supplementary Figure 2. Analysis of the most frequently mutated genes at time of first malignancy (t0: TP53, DNMT3A, TET2, ASXL1).** **A)** Median variant allele frequency (VAF) between t0 and t-MN, with focus (**B**) on the genes with significant VAF increase. **2C** Type of mutations. **2D** *TP53*-driven CHIP outcome according to VAF at t-MN.


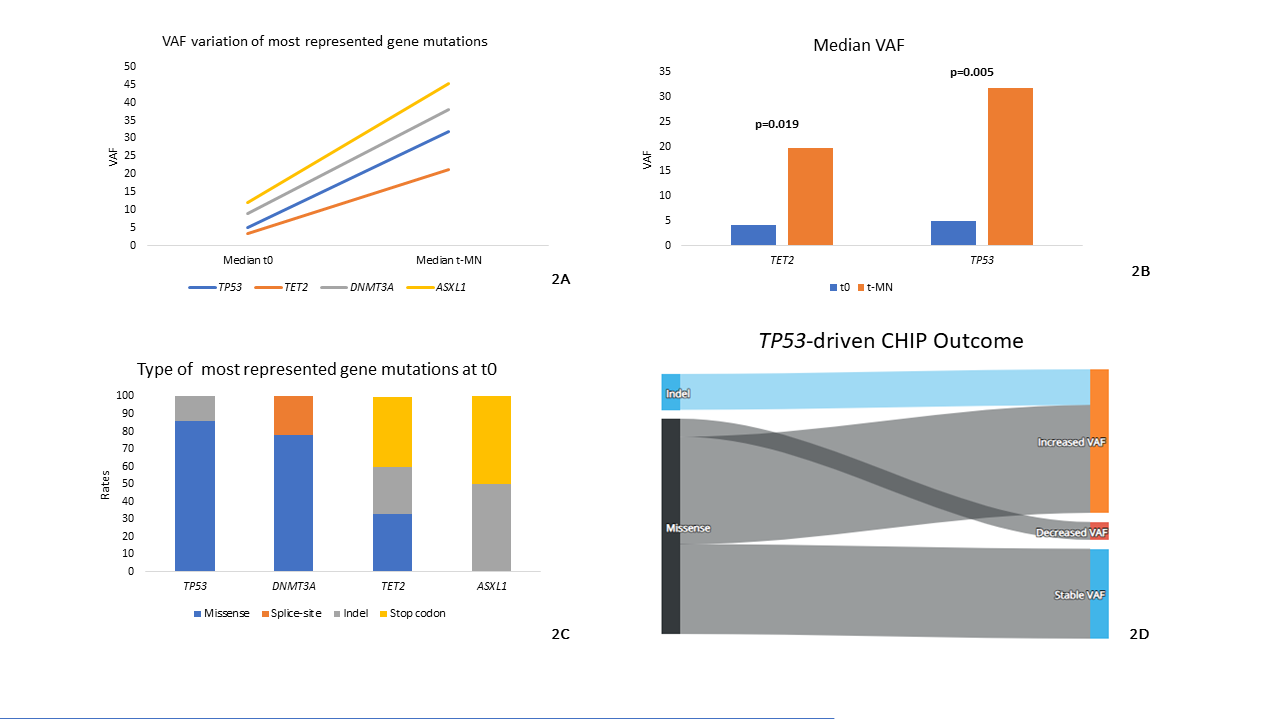


**Supplementary Figure 3. Correlations between main CHIP driver gene at time of first malignancy and emergence of new mutations at t-MN: TET2 (3A), DNMT3A (3B) and ASXL1 (3C).**


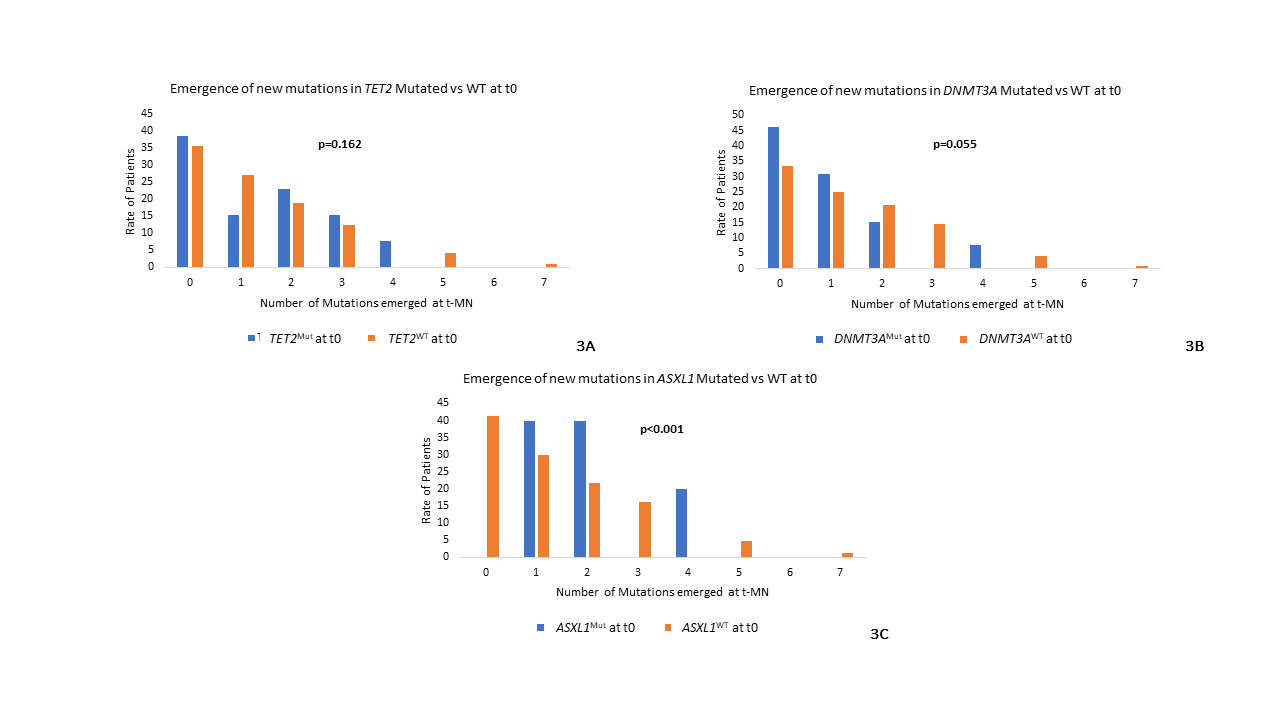


**Supplementary Figure 4. Karyotype analysis at t-MN. 4A** Landscape of karyotype abnormalities. **4B/C/D** Significant correlations between karyotype abnormalities and mutations.


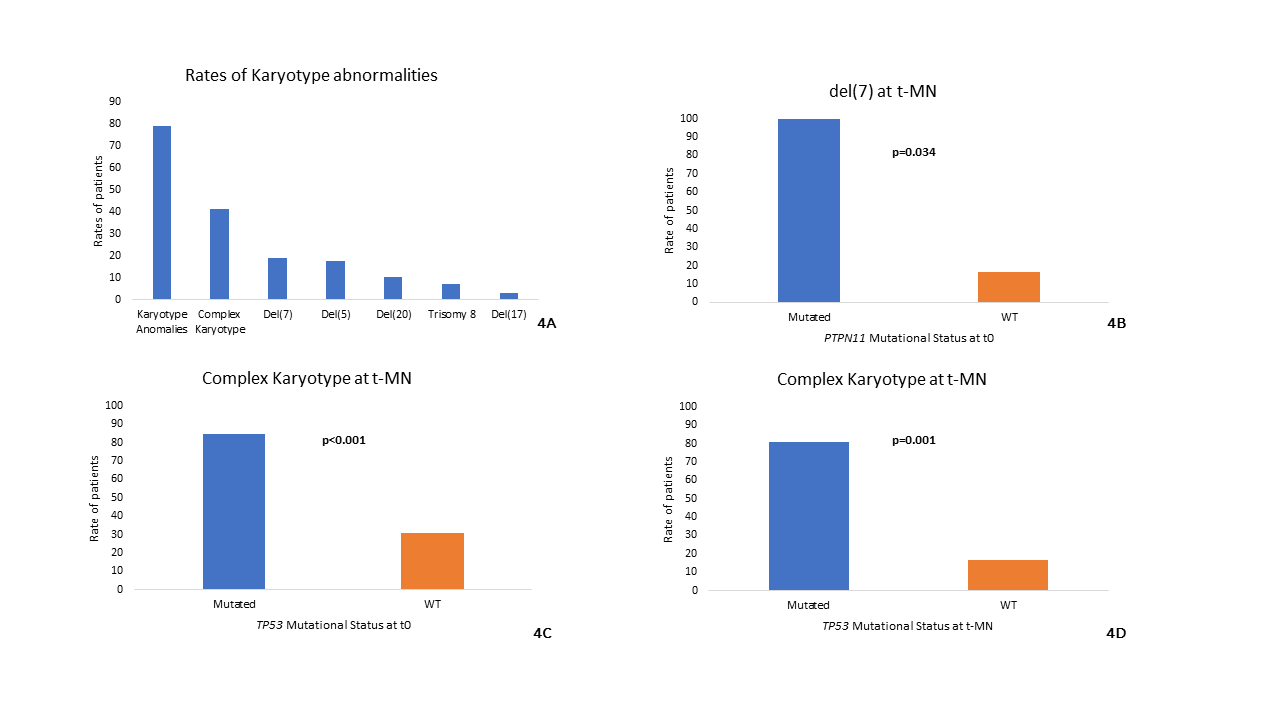


**Supplementary Figure 5. Analysis of t-MN with normal karyotype (NK).** **A)** Number of mutations and number of patients carrying the mutations at the time of first tumor (t0) are represented in blue shaded columns. Number of mutations and number of patients carrying the mutations at the time of t-MN are represented in red shaded columns. **B)** Landscape of mutations emerged at t-MN. **C)** Number of mutations at t0 in patients developing NK t-MN (blue columns) and abnormal karyotype t-MN (orange columns).


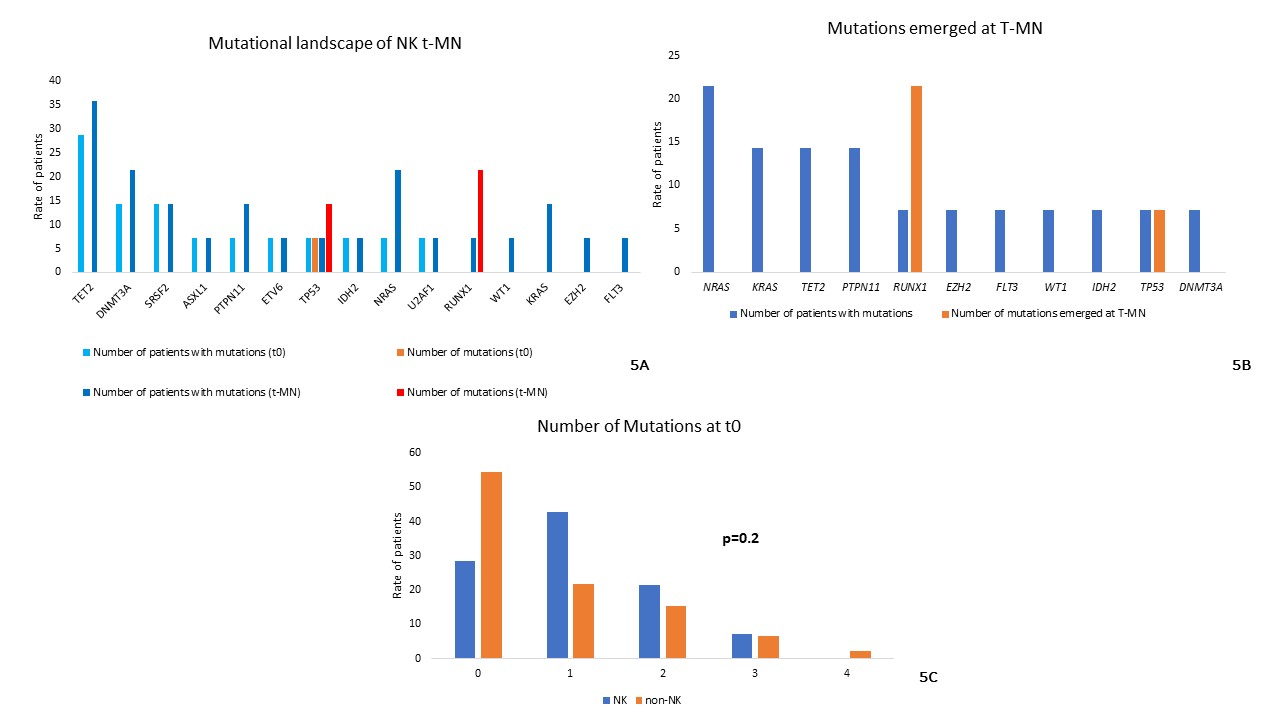


**References**

1 Gibson CJ, Lindsley RC, Tchekmedyian V, Mar BG, Shi J, Jaiswal S *et al.* Clonal Hematopoiesis Associated With Adverse Outcomes After Autologous Stem-Cell Transplantation for Lymphoma. *J Clin Oncol Off J Am Soc Clin Oncol* 2017; **35**: 1598–1605.

2 Xie Z, Lasho T, Khurana A, Ferrer A, Finke C, Mangaonkar AA *et al.* Prognostic relevance of clonal hematopoiesis in myeloid neoplastic transformation in patients with follicular lymphoma treated with radioimmunotherapy. *Haematologica* 2023. doi:10.3324/haematol.2023.283727.

3 Voso M-T, Pandzic T, Falconi G, Denčić-Fekete M, De Bellis E, Scarfo L *et al.* Clonal haematopoiesis as a risk factor for therapy-related myeloid neoplasms in patients with chronic lymphocytic leukaemia treated with chemo-(immuno)therapy. *Br J Haematol* 2022; **198**: 103–113.

4 Liu Y, Derkach A, Lewis N, Zhu M, Zhang Y, Arcila M *et al.* Clonal hematopoiesis in diffuse large B-cell lymphoma: clinical impact and genetic relatedness to lymphoma and therapy-related myeloid neoplasm. *Haematologica* 2022. doi:10.3324/haematol.2022.281724.

5 Gramegna D, Bertoli D, Cattaneo C, Almici C, Re A, Belotti A *et al.* The role of clonal hematopoiesis as driver of therapy-related myeloid neoplasms after autologous stem cell transplantation. *Ann Hematol* 2022; **101**: 1227–1237.

6 Khalife-Hachem S, Saleh K, Pasquier F, Willekens C, Tarabay A, Antoun L *et al.* Molecular Landscape of Therapy-related Myeloid Neoplasms in Patients Previously Treated for Gynecologic and Breast Cancers. *HemaSphere* 2021; **5**: e632.

7 Takahashi K, Wang F, Kantarjian H, Doss D, Khanna K, Thompson E *et al.* Preleukaemic clonal haemopoiesis and risk of therapy-related myeloid neoplasms: a case-control study. *Lancet Oncol* 2017; **18**: 100–111.

8 Gillis NK, Ball M, Zhang Q, Ma Z, Zhao Y, Yoder SJ *et al.* Clonal haemopoiesis and therapy-related myeloid malignancies in elderly patients: a proof-of-concept, case-control study. *Lancet Oncol* 2017; **18**: 112–121.

9 Mouhieddine TH, Sperling AS, Redd R, Park J, Leventhal M, Gibson CJ *et al.* Clonal hematopoiesis is associated with adverse outcomes in multiple myeloma patients undergoing transplant. *Nat Commun* 2020; **11**: 2996.

10 Soerensen JF, Aggerholm A, Rosenberg CA, Bill M, Kerndrup GB, Ebbesen LH *et al.* Clonal evolution in patients developing therapy-related myeloid neoplasms following autologous stem cell transplantation. *Bone Marrow Transplant* 2022; **57**: 460–465.
